# Neuroophthalmic Manifestations of Intracranial Tumours in Children

**DOI:** 10.1155/2021/7793382

**Published:** 2021-05-15

**Authors:** Megha Kotecha, Sarang Gotecha, Ashish Chugh, Prashant Punia

**Affiliations:** ^1^Department of Ophthalmology, Dr. D.Y. Patil Medical College and Hospital, Pimpri, Pune, Maharashtra, India; ^2^Department of Neurosurgery, Dr. D.Y. Patil Medical College and Hospital, Pimpri, Pune, Maharashtra, India

## Abstract

**Background:**

All children between 0 and 16 years presenting with brain tumours confirmed by Magnetic Resonance Imaging (MRI) and treated surgically in our institute were included in this study.

**Objective:**

The aim of this study is to evaluate the neuroophthalmic and clinical characteristics of intracranial space occupying lesions in children.

**Methods:**

Neuroophthalmic manifestations along with location of the tumour by contrast-enhanced MRI, type of surgical intervention, and postoperative histopathological diagnosis were evaluated.

**Results:**

In pediatric brain tumours, male preponderance was seen and supratentorial location was more common in general, while in older children, infratentorial tumours were more common than supratentorial tumours. Headache, vomiting, and cerebellar signs were the commonest neurological features. Diminution of vision, diplopia, and strabismus were the commonest ophthalmic symptoms. Papilledema, ophthalmoparesis, and nystagmus were the most frequent ophthalmological signs. Neurological manifestations of seizures, altered sensorium and motor deficits were more frequently seen in supratentorial tumours, while cranial nerve involvement and ataxia were seen in infratentorial tumours. Ophthalmological manifestations including diplopia, strabismus, ophthalmoparesis, and nystagmus were more frequently seen in infratentorial tumours. Astrocytoma was the most frequent histopathological diagnosis followed by medulloblastoma.

**Conclusion:**

Diagnosis of pediatric intracranial tumours is complex and requires a multidisciplinary approach for prompt management. An ophthalmologist should have a high index of suspicion for brain tumours especially in patients presenting with common ocular symptoms like diminution of vision, diplopia, and strabismus without any neurological symptoms.

## 1. Introduction

Central nervous system tumours (CNS) are the most common solid tumours in children and a leading cause of mortality and morbidity in children worldwide [1, 2]. In India, according to the Indian Council of Medical Research, National Cancer Registry data, the incidence of pediatric brain tumours is around 2% [2].

Despite advances in neuroimaging, early diagnosis of pediatric CNS tumours remains a dilemma. Contributing factors to this problem are varied presentation and rarity of these tumours in childhood [3]. The frequent presentation of brain tumours in children includes features of raised intracranial pressure like headache and vomiting. However, in about 10% of affected children, visual symptoms can be the mode of presentation [4]. A lack of awareness of the ophthalmic signs and symptoms of brain tumours can lead to a delayed diagnosis and their management.

Visual manifestations of brain tumours in children include blurring of vision, diplopia, ocular nerve palsy, papilledema, optic atrophy, ptosis, nystagmus, strabismus, proptosis, and photophobia. [5] As the visual system in children is still developing, brain tumours and their surgical management can alter the normal neuroanatomical structures of the visual system and cause permanent visual impairment [6].

The purpose of this study is to evaluate the neuroophthalmic and clinical characteristics of intracranial-space-occupying lesions in children.

## 2. Materials and Methods

This prospective observational study was carried out for a period of 2.5 years from June 2017 to December 2019 after Ethical Committee Clearance. All children between 0 and 16 years presenting with brain tumours confirmed by Magnetic Resonance Imaging (MRI) and treated surgically in our institute were included in this study. Patients who had received chemotherapy or radiotherapy before surgery or with coexistent primary neoplasm elsewhere were excluded from the study. Patient's age; gender; neurological symptoms which include history of headache, nausea/vomiting, seizures, and anosmia; and neurological signs which include cerebellar signs, speech involvement, motor deficits, altered sensorium, endocrine involvement, and cranial nerve palsies were evaluated by the neurosurgical team.

Ophthalmic features which include diminution of vision and visual field defects, ocular pain, red eye, proptosis, papilledema, disc pallor, shunting of vessels, ptosis, pupil abnormality, nystagmus, and strabismus were evaluated by the ophthalmologist.

Other parameters which were evaluated were the location of the tumour by contrast-enhanced MRI, type of surgical intervention, and postoperative histopathological diagnosis.

## 3. Results

A total of 35 patients were evaluated. Ages ranged from 8 months to 16 years, and the mean age of the patients was 8.28 years. The average duration of symptoms in our study was 6.45 weeks.

According to the location, 16 cases were infratentorial in location and 19 cases were supratentorial in location. Frequency of neurological signs and symptoms according to supra- and infratentorial locations are mentioned in Table 1.

23 patients presented with ocular symptoms at the time of presentation, whereas 31 patients were found to have abnormal findings after detailed ophthalmic evaluation. The ophthalmic manifestations of the patients according to infratentorial and supratentorial locations are illustrated in Table 2.

commonest ophthalmic symptoms were diminution of vision and diplopia, and the most common ophthalmic sign was papilledema followed by ophthalmoparesis and nystagmus.

The location of the tumours according to their age distribution is illustrated in Table 3. Cerebellar tumours were highest in number, followed by sellar region tumours. Out of the 6 sellar region tumours, 1 case was a sellar tumour, 2 cases had a suprasellar extension, 1 case had a suprasellar and parasellar extension, and 1 case was parasellar in location. Out of the 5 ventricular tumours, 3 cases were in the left lateral ventricle, and 1 case each was in the third ventricle and the 4th ventricle.

### 3.1. Operative Characteristics

The type of surgical interventions are mentioned in Table 4. Surgical resection of the tumours was done in 28 (80%) patients, CSF diversion procedure was done in 4 (11.4%) patients, stereotactic biopsy of the patient was done in 1 (2.9%) patient, and primary radiation therapy was advised in 2 (5.7%) patients.

The histopathological diagnosis of all the patients and their distribution according to their location are mentioned in Table 5. The most common histopathological diagnosis was astrocytoma followed by medulloblastoma.

## 4. Discussion

Pediatric brain tumours are a heterogenous group of neoplasms which vary in their cell of origin, mode of presentation, management protocols, and clinical outcomes [2]. Treatment of pediatric CNS neoplasms requires a multidisciplinary team which includes a neurooncologist, a neuroradiologist, a neuroophthalmologist, a neurosurgeon, and a pediatric neurologist. Survival rate in these cases have improved over the years as a result of a multidisciplinary approach, improvements in diagnostic modalities, surgical technologies, adjuvant radiotherapy and chemotherapy, and surveillance techniques for tumour recurrence [7].

This study was undertaken to observe a particular cohort of children who were clinically and radiologically diagnosed with intracranial tumours managed by the neurosurgical team along with ophthalmological examination done by the ophthalmology team.

In this study, there were 35 patients with a mean age of 8.28 years which was comparable to the study by Liu et al. [6] and Mehrazin and Yavari [8]. Fifteen patients were girls and 20 were boys. Many studies in the literature on the incidence of intracranial tumours especially studies which include adult patients show female preponderance [9, 10]. This is due to a higher incidence of meningiomas and pituitary adenomas in females among adult patients. In contrast to this observation, the literature on pediatric brain tumours suggests a higher incidence of intracranial tumours in boys similar to our study [11, 12]. In developing countries, this may also be a reflection of the prevalent gender bias of the relatives seeking treatment.

The road to diagnosis of brain tumours can be a potential challenge as many clinical symptoms can mimic other common childhood ailments. The clinical signs of the tumour can vary based on the location, age, and aggressiveness of the tumour, and a high index of suspicion followed by prompt evaluation and surgical intervention is required, the delay of which can lead to untoward complications [13]. Neurological symptoms produced by intracranial tumours can be local or general. General symptoms can result from a progressive enlargement of the tumour within the limited volume of the cranial vault causing raised intracranial pressure and related symptoms, whereas local symptoms are due to the effects of the tumour on contiguous areas of the brain [14].

The commonest neurological symptom was headache followed by nausea and vomiting which confirms the well-known predominance of symptoms of raised intracranial pressure in these cases according to the literature [2, 3, 10, 14]. The higher incidence of headache in our study can be attributed to the delay in approaching a specialist and reaching a diagnosis both of which are predominant in developing countries. In our view, as we are practicing in a tertiary care centre, more often than not, patients with complications or those requiring complex surgeries are referred to our centre which is another cause for the late presentation of cases. Intracranial tumours can cause raised intracranial pressure by various mechanisms which include increased brain volume due to the tumour itself, vasogenic edema due to the tumour, mass effect on the ventricular system leading to obstructive hydrocephalus, compression or invasion of the venous system by the tumour, and meningeal involvement of the tumour [1].

The other neurological signs and symptoms seen in our study were cerebellar signs (45.7%), optic nerve involvement (82.9%), other types of cranial nerve involvement (42.8%), seizures (40%), altered sensorium (14.3%), ataxia (11.4%), motor deficits (8.6%), endocrine involvement (5.7%), and increased head circumference (2.9%). These are consistent with various similar studies in the literature on pediatric intracranial tumours [2, 3, 11, 12].

Brain tumours can alter the normal neuroanatomical structures of the visual system leading to visual impairment and dysfunction which can have a lifelong impact on the quality of life of patients and their families [6]. Ophthalmological examination in these cases are not only beneficial for assessing the visual status of these patients but also helpful for prognosticating the patients and their families regarding the visual outcome which in some cases can also affect decision-making in the selection of the type of intervention in these cases.

Ophthalmic signs and symptoms are a mode of presentation in children with intracranial tumours, and the ophthalmological evaluation must be performed with greater attention as the presentation in children is less specific than that in adults [11].

Neuroophthalmologic evaluation should be done by protocol for children with brain tumours. However, early assessment of vision may not be done routinely at all hospitals due to various reasons which include (a) priority given to brain tumours, (b) lack of apparent visual symptoms at the time of presentation, (c) inability to clearly describe symptoms which is particularly more common in young children, (d) difficulty in examining young children, and (e) difficult access to a neuroophthalmologist [1].

Out of the 35 patients in our study, 23 (65.71%) patients presented with ocular symptoms and 31 (88.57%) patients were found to have abnormal findings after detailed ophthalmological examination. Ocular symptoms at the time of presentation are higher than in some studies in the literature like the study by Liu et al. which can again be attributed to the late presentation of these cases in developing countries [6]. However, in the same study, the authors report the incidence of 90% of abnormal findings on ophthalmological examination which is similar to our study.

The visual symptoms seen in our study were diminution of vision, diplopia, strabismus, and ptosis. All these visual symptoms can be devastating and lead to long-term effects on the quality of life for the patient and the family. Visual impairment can be caused by affecting both the afferent and the efferent pathways. Direct compression of the visual pathway by the tumour can lead to diminution of vision, visual field defect, and ocular motility defects [13]. Furthermore, obstructive hydrocephalus, mass effect of the brain tumour, cerebral odema, and leptomeningeal involvement by the tumour can cause raised intracranial pressure eventually leading to papilledema, pale disc, and loss of vision. In our study, 13 (37.1%) patients presented with diminution of vision out which 6 patients of sellar region tumours had direct compression on the visual pathway, whereas in 7 patients, vision loss was secondary to raised intracranial pressure. Strabismus was present in 12 (34.3%) patients out of which 11 patients had infratentorial tumours and 1 patient had a supratentorial tumour, which is again consistent with the literature which states that strabismus is more common in posterior fossa tumours [6]. Ptosis was seen in 3 (8.6%) children which included 2 patients of pontine tumours and 1 case of sellar tumour with parasellar extension.

In their study on children diagnosed with intracranial-space-occupying lesions, Alswaina et al. have highlighted the role of an ophthalmologist in these cases. They have reported an incidence of optic atrophy in 46%, diminution of vision in 46%, papilledema in 24%, nystagmus in 24%, sixth nerve palsy in 19%, and third nerve palsy in 12% [4]. These findings were comparable to our study.

Ophthalmoparesis was seen in 15 (42%) patients in our study. Palsies of III, IV, and VI nerve were more common in children with infratentorial tumours than in children with supratentorial tumours which again correlated with the data available in the literature [15]. Acquired third nerve and fourth nerve palsies were seen in 3 cases including 2 cases of pontine tumours and one case of a sellar tumour with suprasellar and parasellar extension which were all due to direct compression on the nerves in all 3 cases. Acquired sixth nerve palsy was seen in 15 (42.9%) cases in our study out of which direct compression was seen in 6 cases and indirect compression due to posterior fossa tumours was seen in 9 cases. In patients presenting with ophthalmoparesis, after ruling out acquired palsies of the third, fourth, and sixth cranial nerves, other etiologies including myasthenia gravis, local inflammation/infection, migraine, and local infarction should be ruled out [4].

Ophthalmological signs seen in our study were papilledema, nystagmus, disc pallor, visual field defects, and pupillary abnormality. Papilledema or pale disc from pediatric brain tumours can result from tumours intrinsic to the optic nerves or optic chiasm, tumours contiguous to the optic nerve or chiasm, or tumours located elsewhere causing nonlocalizing increased intracranial pressure [4]. In our study, papilledema was seen in 17 (48.6%) cases and disc pallor was seen in 11 (31.4%) cases. Nystagmus was seen in 14 cases of which 10 cases were posterior fossa tumours, 3 cases were pontine tumours, and 1 case was pineal gland tumour.

In our study, visual field defects were present clinically and confirmed by perimetry as bitemporal hemianopia in 2 patients of sellar region tumours. However, 4 patients had clinical symptoms of field defects which could not be confirmed by perimetry due to noncompliance of the children. In a study carried out by Harbert et al., the authors reported that 15.2% of children with CNS tumours have visual field defects that go unrecognized and recommended that serial neuroophthalmological evaluation of children with brain tumours is required to diagnose a visual field defect since patient or caregiver reporting may be limited [16].

Pupillary abnormality was seen in 4 patients (11.4%) out of which 2 patients were cases of pineal region tumours, and these patients presented with features of Perinaud's syndrome which include upgaze palsy convergence-retraction nystagmus, light-near dissociation of pupils, and lid retraction [5].

After complete neurological and ophthalmic evaluation of the patient, imaging was done in all patients with suspected intracranial tumours. Contrast-enhanced MRI of the brain is the investigation of choice which was done in all our cases. Additionally, depending on the type and location of the tumour, MRI angiogram to display vasculature alongside the tumour, MR spectroscopy to assess the metabolic activity of the tumour, and functional MRI and tractography to provide additional information on the course of important white matter tracts and their relationship with the tumour were done for planning of surgery. Sedation was required in younger children or children not cooperative to undergo MRI.

In our study, 20% of the cases were located in the cerebral cortex, 31.4% of the cases were located in the cerebellum, 8.6% of the cases were located in the brainstem, 17.1% of the cases were located in the pituitary and suprasellar region, and 14.2% of the cases were intraventricular in location. According to the literature, about 25-30% are in a supratentorial location, followed by cerebellum (15-20%), brainstem (10-12%), pituitary and suprasellar regions (10-15%), and brain ventricles (5-6.4%) which is comparable to our study [17, 18].

Of the 35 tumours, 19 (54.3%) were supratentorial and 16 (45.7%) were infratentorial. This was comparable to the study carried out by Suresh . [13] Neurological and visual symptoms according to their locations are listed in Table 3. In our study, incidence of neurological features including seizures, altered sensorium, and motor deficits are higher in supratentorial tumours, whereas incidence of cranial nerve involvement and ataxia were higher in infratentorial tumours. Incidence of ophthalmological manifestations including diplopia, strabismus, ophthalmoparesis, and nystagmus are higher in infratentorial tumours. All these findings are comparable to the study on clinical presentation of supratentorial and infratentorial intracranial tumours in pediatric patients by Sánchez-Sánchez . [19]

In older children, infratentorial tumours are more common than supratentorial tumours [4, 20]. Consistent with the literature, in our series, 11 of 16 patients with posterior fossa tumours were older children. Ophthalmological signs and symptoms were present in 15 out of 16 patients (93.75%). This is higher in comparison to a study of posterior fossa tumours by Gadgil ., the reason for which can be attributed to the retrospective nature of their study and neglect of ocular presentation at initial evaluation as reported by the authors [21]. In our prospective study, detailed ophthalmological examination was done at the time of presentation by the ophthalmology team. Neurological presentation of posterior fossa tumours were as follows: headache and cerebellar signs, which were present in all the patients; nausea and vomiting, which were present in 14 (87.5%) of the cases; nystagmus, which was present in 13 (81.25%) of the cases; strabismus, which was seen in 11 (68.75%) of the cases; and papilledema, which was seen in 9 (56.25%) of the cases.

Out of the 16 cases of posterior fossa tumours in our study, medulloblastoma (6 cases) was the commonest tumour followed by astrocytoma (5 cases). This is in contrast to most studies on pediatric posterior fossa tumours which state that the incidence of astrocytoma is higher than that of medulloblastoma in these cases [22, 23]. The other posterior fossa tumours seen in our study were arachnoid cyst (3 cases), giant cell tumour (1 case), and infected epidermoid cyst (1 case).

Six cases of sellar region tumours were seen in our study, out of which 3 cases were craniopharyngiomas, one case each of pituitary adenoma, arachnoid cyst, and optic nerve glioma. This collaborates with the study of Laws and Sheehan, who have suggested that diagnosis of sellar region tumours in pediatric patients requires a multidisciplinary approach and detailed ophthalmic and neurological tests are critical in these cases [24].

Surgical resection is the main modality of treatment for most pediatric brain tumours. Depending on the tumour type, the goals of surgical management are to achieve a tissue diagnosis and reestablish normal CSF (cerebrospinal fluid) pathways, CSF diversion, tumour debulking, and complete tumour resection [25]. In accordance with these principles, craniectomy with surgical resection was done in 22 cases and CSF diversion was done in 4 cases, out of which ventriculoperitoneal shunt was done in 3 cases and cystoperitoneal shunt was done in 1 case; endoscopic procedure was done in 6 cases, out of which endoscopic transsphenoidal excision was done in 3 cases, endoscopic tumour excision was done in 2 cases, and endoscopic biopsy was done in 1 case; stereotactic biopsy was done in 1 case; and a patient was referred for primary radiotherapy in 2 cases.

Astrocytoma was the most frequent tumour seen in our study followed by medulloblastoma. This is consistent with most studies in the literature [2, 6, 15, 26–28].

The frequency of craniopharyngioma (11.4%) in the present study was high in our study but was comparable to various studies from India, Egypt, Japan, and Brazil [29–34]. Kassam et al. had a classification system for craniopharyngiomas based on the infundibulum to strategize the surgical approach. Type I tumours are preinfundibular, which are subchiasmatic tumours displacing the optic chiasm superiorly and posteriorly. Type II tumours are transinfundibular, which can extend into the third ventricle. Type III is retroinfundibular, which can either extend superiorly into the third ventricle or inferiorly into the pontine cisterns [35]. Type IV tumours are primarily located in the third ventricles. According to this classification, there was 1 case each of type I and type IV and there were 2 cases of type II.

The other tumours present in our study include pineoblastoma, meningioma, choroid plexus papilloma, pituitary adenoma, and tuberculoma which are comparable to various studies in the literature [4, 29, 33, 34, 36].

## 5. Limitation of the Study

Statistical significance was not evaluated because of the very small sample size of the study.

## 6. Conclusions

To conclude, diagnosis of pediatric intracranial tumours is complex and requires a multidisciplinary approach for prompt management. Neuroophthalmic manifestations are common but can be easily missed in children with intracranial tumours. With increased survival rates, pediatric intracranial tumours with ophthalmic involvement can cause devastating long-term consequences due to vision loss or ocular motor abnormalities eventually leading to decreased quality of life.

An ophthalmologist should have a high index of suspicion for brain tumours especially in patients presenting with common ocular symptoms like diminution of vision, diplopia, and strabismus without any neurological symptoms. Ophthalmological evaluation and monitoring can also be helpful in these cases for preoperative planning and systematic treatment monitoring and for postoperative prognostication and multidisciplinary care of the patient.

We also conclude that in any child presenting to an ophthalmologist with additional signs and symptoms that could result from a CNS tumour, a thorough neuroophthalmic examination should be done. This can help in early diagnosis and prompt management of pediatric intracranial tumours and also helps in improvement of visual prognosis. Other salient features observed and concluded in our study include the following:

1. In pediatric brain tumours, male preponderance is seen which collaborates with many studies in the literature. Supratentorial location was more common in general, while in older children, infratentorial tumours are more common than supratentorial tumours
2. Headache, vomiting, and cerebellar signs were the commonest neurological features; diminution of vision, diplopia, and strabismus were the commonest ophthalmic symptoms; while papilledema, ophthalmoparesis, and nystagmus were the most frequent ophthalmological signs
3. Neurological manifestations of seizures, altered sensorium, and motor deficits were more frequently seen in supratentorial tumours, while cranial nerve involvement and ataxia were seen in infratentorial tumours. Ophthalmological manifestations including diplopia, strabismus, ophthalmoparesis, and nystagmus were more frequently seen in infratentorial tumours
4. Astrocytoma was the commonest histopathological diagnosis followed by medulloblastoma, which again collaborated with various studies in the literature

## Figures and Tables

**Table 1:** Frequency of neurological signs and symptoms according to supratentorial and infratentorial location of the tumour.

Clinical features	Infratentorial	Supratentorial	Total
Headache	16 (45.7%)	17(%)	33 (94.3%)
Nausea/vomiting	14 (40%)	10 (28.6%)	24 (68.6%)
Ophthalmic involvement	15 (42.9%)	15 (42.9%)	30 (85.7%)
Cerebellar signs	—	16 (45.7%)	16 (45.7%)
Ophthalmoparesis			15 (42.8%)
Cranial nerve II	14	15	29 (82.9%)
Cranial nerve III	2	1	3 (8.6%)
Cranial nerve IV	2	1	3 (8.6%)
Cranial nerve V	1	0	1 (2.9%)
Cranial nerve VI	13	2	15 (42.9%)
Cranial nerve VII	4	0	4 (11.4%)
Cranial nerve VIII	1	0	1 (2.9%)
Lower cranial nerves	2	—	2 (5.7%)
Seizures	1 (2.9%)	13 (37.1%)	14 (40%)
Altered sensorium	1 (2.9%)	4 (11.4%)	5 (14.3%)
Ataxia	4 (11.4%)	0	4 (11.4%)
Motor deficit	0	3 (8.6%)	3 (8.6%)
Endocrine involvement (stunted growth)	0	2 (5.7%)	2 (5.7%)
Increased head circumference	—	1 (2.9%)	1 (2.9%)

**Table 2:** Ophthalmic manifestations of the tumours according to infratentorial location and supratentorial location of the tumour.

	Infratentorial	Supratentorial	Total
*Ophthalmic symptoms*			
Visual acuity involvement	6 (17.1%)	7 (20%)	13 (37.1%)
Diplopia	11	2 (5.7%)	13 (37.1%)
Strabismus	11	1 (2.9%)	12 (34.3%)
Ptosis	2 (5.7%)	1 (2.9%)	3 (8.6%)
*Ophthalmic signs*			
Papilledema	9 (25.7%)	8 (22.9%)	17 (48.6%)
Ophthalmoparesis	13 (37.1%)	2 (5.7%)	15 (42.8%)
Nystagmus	13 (37.1%)	1 (2.9%)	14 (40%)
Disc pallor	5	6 (17.1%)	11 (31.4%)
Pupillary abnormality	2 (5.7%)	2 (5.7%)	4 (11.4%)

**Table 3:** Tumour location according to age distribution.

Tumour location	0-5 years	6-10 years	11-16 years	Total
Cerebellar tumour	1	5	5	11 (31.4%)
Sellar region tumours	0	3	3	6 (17.14%)
Ventricular tumours	3	1	1	5 (14.3%)
Left frontal lobe tumour	0	1	2	3 (8.6%)
Pontine tumour	2	1	0	3 (8.6%)
Frontoparietal tumour	2	0	0	2 (5.7%)
Left basal ganglia tumour	0	0	1	1 (2.9%)
Multiple intracranial tumour	0	0	1	1 (2.9%)
Pineal gland tumour	1	0	0	1 (2.9%)
Prepontine tumour	1	0	0	1 (2.9%)
Right frontotemporoparietal tumour	1	0	0	1 (2.9%)
Total	11	11	13	35

**Table 4:** Type of surgical intervention.

Operative characteristics	Frequency	Percentage
Craniectomy with surgical resection	22	62.9%
CSF diversion	4	11.4%
Transsphenoidal gross total resection	3	8.6%
Endoscopic gross total tumour excision	2	5.7%
Primary radiotherapy	2	5.7%
Endoscopic biopsy	1	2.9%
Stereotactic biopsy	1	2.9%
Total	35	100.0%

**Table 5:** Histopathological diagnosis and their distribution according to their location.

Histopathology	Frequency	Location	Frequency
Medulloblastoma	6 (17.1%)	Cerebellum	6
High-grade glioma	5 (14.3%)	Pons	3
		Left frontal lobe	1
		Right frontoparietal lobe	1
Low-grade glioma	4 (11.4%)	Cerebellum	2
		Left frontal lobe	1
		Left lateral ventricle	1
Abscess	4 (11.4%)	Bilateral frontoparietal lobe	1
		Left frontoparietal lobe	1
		Left basal ganglia	1
		Multiple intracranial lesions	1
Arachnoid cyst	4 (11.4%)	4th ventricle	1
		Cerebellum	2
		Sellar region	1
Craniopharyngioma	4 (11.4%)	Sellar region	3
		Third ventricle	1
Choroid plexus papilloma	1 (2.9%)	Left lateral ventricle	1
Giant cell tumour	1 (2.9%)	Cerebellum	1
Infected epidermoid cyst	1 (2.9%)	Prepontine region	1
Meningioma	1 (2.9%)	Left lateral ventricle	1
Optic nerve glioma	1 (2.9%)	Sellar region	1
Pineoblastoma	1 (2.9%)	Pineal region	1
Pituitary adenoma	1 (2.9%)	Sellar region	1
Tuberculoma	1 (2.9%)	Left frontal lobe	1
Total	35		35
